# Pulmonary inflammation and tumor induction in lung tumor susceptible A/J and resistant C57BL/6J mice exposed to welding fume

**DOI:** 10.1186/1743-8977-5-12

**Published:** 2008-09-08

**Authors:** Patti C Zeidler-Erdely, Michael L Kashon, Lori A Battelli, Shih-Houng Young, Aaron Erdely, Jenny R Roberts, Steven H Reynolds, James M Antonini

**Affiliations:** 1 Pathology and Physiology Research Branch, Health Effects Laboratory Division, National Institute for Occupational Safety and Health, Morgantown, USA; 2 Biostatistics and Epidemiology Branch, Health Effects Laboratory Division, National Institute for Occupational Safety and Health, Morgantown, USA; 3 Toxicology and Molecular Biology Branch, Health Effects Laboratory Division, National Institute for Occupational Safety and Health, Morgantown, USA

## Abstract

**Background:**

Welding fume has been categorized as "possibly carcinogenic" to humans. Our objectives were to characterize the lung response to carcinogenic and non-carcinogenic metal-containing welding fumes and to determine if these fumes caused increased lung tumorigenicity in A/J mice, a lung tumor susceptible strain. We exposed male A/J and C57BL/6J, a lung tumor resistant strain, by pharyngeal aspiration four times (once every 3 days) to 85 μg of gas metal arc-mild steel (GMA-MS), GMA-stainless steel (SS), or manual metal arc-SS (MMA-SS) fume, or to 25.5 μg soluble hexavalent chromium (S-Cr). Shams were exposed to saline vehicle. Bronchoalveolar lavage (BAL) was done at 2, 7, and 28 days post-exposure. For the lung tumor study, gross tumor counts and histopathological changes were assessed in A/J mice at 48 and 78 weeks post-exposure.

**Results:**

BAL revealed notable strain-dependent differences with regards to the degree and resolution of the inflammatory response after exposure to the fumes. At 48 weeks, carcinogenic metal-containing GMA-SS fume caused the greatest increase in tumor multiplicity and incidence, but this was not different from sham. By 78 weeks, tumor incidence in the GMA-SS group versus sham approached significance (p = 0.057). A significant increase in perivascular/peribronchial lymphoid infiltrates for the GMA-SS group versus sham and an increased persistence of this fume in lung cells compared to the other welding fumes was found.

**Conclusion:**

The increased persistence of GMA-SS fume in combination with its metal composition may trigger a chronic, but mild, inflammatory state in the lung possibly enhancing tumorigenesis in this susceptible mouse strain.

## Background

For decades researchers have tried to determine whether exposure to welding fumes poses an increased risk of lung cancer. Indeed, this health concern is one of the most important questions in welding fume-related toxicological research. The International Agency for Research on Cancer has deemed welding fume a group 2B agent, defined as a mixture "possibly carcinogenic" to humans [1]. This categorization of welding fume carcinogenicity, however, was based on limited evidence in humans and a lack of animal data.

The harmful health effects of welding are well documented and epidemiological evidence generally supports the hypothesis that exposure to welding fume increases lung cancer risk, but confounders such as asbestos exposure and smoking obscure these findings [2-5]. Debate also exists over which type of welding may pose the greater risk. Interestingly, fumes from both non-carcinogenic metal-containing mild steel (MS) and carcinogenic metal-containing stainless steel (SS) welding wire have been shown to increase lung cancer risk in welders [6,7]. For these reasons, we initiated a multipart study to ultimately determine the carcinogenic potential of SS and MS welding fumes in an animal model.

Electric arc welding joins pieces of metal that are rendered liquid by heat. Arc temperatures above 4000°C heat the base metal pieces to be joined and the consumable electrode wire that is continuously fed into the weld. The vaporized metals, derived primarily from the wire, react with air and form the fume, which consists of a complex mixture of metal oxides. Depending on the welding process employed, the electrode coating, shielding gases, fluxes, base metal, and paint or surface coatings also may comprise the welding fume [8].

Among the numerous types of welding processes, manual metal arc (MMA) and gas metal arc (GMA) welding are two types commonly used in the workplace. Welding processes that use SS wire produce fumes that contain carcinogenic metals such as chromium (Cr) and nickel (Ni). Welding fume from MS wire primarily consists of iron (Fe) with a lesser amount of manganese (Mn), but no Cr or Ni. In addition, fumes from MMA-SS wire yield more water-soluble metals in suspension than GMA-SS or GMA-MS fumes and consequently, in animal models, induced a more potent acute lung toxicity [9,10].

In this investigation, we used lung tumor susceptible A/J mice, a common animal model for lung carcinogenesis studies. Compared to the essentially lung tumor resistant C57BL/6J strain, A/J mice exhibit high susceptibility to spontaneous and chemically induced lung tumors [11]. Further, the lung tumors in the A/J mouse display many morphological, histopathological, and molecular similarities to human pulmonary adenocarcinomas, which makes them a relevant model for lung cancer research [12,13].

Our first objective was to characterize the potential of welding fumes of different metal compositions, or components thereof, to cause acute lung toxicity in lung tumor susceptible (A/J) and resistant (C57BL/6J) mice. We rationalized that this direct strain comparison would be invaluable for interpretation of our second objective which was to determine the tumorigenic potential of these different welding fumes in the susceptible A/J mouse model.

## Results

### Bronchoalveolar lavage (BAL) findings 2 days after exposure: GMA-MS and GMA-SS

GMA-MS welding fume caused a similar degree of lung cell death (measured as lactate dehydrogenase activity [LDH]) in both strains and no significant epithelial damage (measured as albumin) compared to the corresponding sham (Table 1). The recovered BAL % polymorphonuclear leukocytes (PMN) was two-fold greater in the A/J compared to the C57BL/6J strain while lymphocytes were not elevated in response to this fume in either strain. GMA-SS exposure was more toxic to the A/J than the GMA-MS fume. Significant lung epithelial damage and cell death occurred in both strains with the A/J having a ~1.7 fold greater response. The %PMN was markedly higher in the A/J strain (44%) versus the C57BL/6J (9%) and lymphocytes were equally elevated in both strains. No strain differences were found for increased macrophage/monocytes in the BAL, but a significant increase in number was observed for the C57BL/6J strain following GMA-SS exposure only (data not shown).

**Table 1:** Bronchoalveolar lavage parameters 2 days post-exposure

Mouse Strain	*n*	Exposure	%Albumin^§^	%LDH^§^	%Lymphocytes^||^	%PMN^||^
A/J	7	GMA-MS	126 ± 4	155 ± 7*	0.8 ± 0.4 (0.2 ± 0.13)	24 ± 3*^‡ ^(1.1 ± 1.1)
C57BL/6J	7	GMA-MS	109 ± 3	134 ± 10*	1.3 ± 0.8 (0.3 ± 0.1)	12 ± 3* (0.05 ± 0.05)
						
A/J	7	GMA-SS	229 ± 13*^†‡^	307 ± 12*^†‡^	2.2 ± 0.8*^† ^(0.2 ± 0.13)	44 ± 3*^†‡ ^(1.1 ± 1.1)
C57BL/6J	6	GMA-SS	139 ± 13*^†^	176 ± 16*	2.1 ± 0.2* (0.3 ± 0.1)	9 ± 2* (0.05 ± 0.05)
						
A/J	7	MMA-SS	331 ± 26*^†‡^	343 ± 33*^†‡^	0.1 ± 0.06 (0.06 ± 0.06)	41 ± 4*^†‡ ^(0.7 ± 0.6)
C57BL/6J	7	MMA-SS	218 ± 16*	230 ± 25*	1.6 ± 0.24*^‡ ^(0 ± 0)	9 ± 2* (0.2 ± 0.08)
						
A/J	7	S-Cr	209 ± 10*	178 ± 14*	0.5 ± 0.16*^† ^(0.06 ± 0.06)	11 ± 0.8* (0.7 ± 0.6)
C57BL/6J	7	S-Cr	266 ± 25*^†‡^	182 ± 20*	2 ± 0.6*^‡ ^(0 ± 0)	9 ± 3* (0.2 ± 0.08)

*Significantly different from corresponding sham; ^†^Between exposed groups of the same mouse strain; ‡Between mouse strains of the same exposure group (p < 0.05)

^§^LDH and albumin data are represented as % corresponding sham.

^||^Percentage from a differential cell count of > 300 cells with the corresponding sham % in parentheses. *Note*. Data are means ± SE. Statistical comparisons were MMA-SS and S-Cr or GMA-MS and GMA-SS. Abbreviations are GMA-MS-gas metal arc-mild steel, GMA-SS-gas metal arc stainless steel, or MMA-SS-manual metal arc stainless steel welding fume; S-Cr-soluble chromium VI; LDH-lactate dehydrogenase; PMN-polymorphonuclear leukocytes

### BAL findings 2 days after exposure: MMA-SS and soluble chromium (S-Cr)

At 2 days post-exposure, both mouse strains had significant lung cell death and epithelial damage following MMA-SS or S-Cr exposure (Table 1). MMA-SS fume was a greater inducer of cell death in the A/J and this was also significantly greater in comparison to the MMA-SS-exposed C57BL/6J strain (~1.5 fold). LDH levels between the MMA-SS and S-Cr exposures were not different in the C57BL/6J. The A/J had significantly greater epithelial damage following MMA-SS exposure compared to the C57BL/6J strain as shown by the increased albumin in the BAL. The C57BL/6J exhibited more epithelial damage than the A/J strain following S-Cr exposure although this was not a consistent finding for other markers of toxicity in this study. Both exposures caused a significant increase in %PMN at 2 days in both mouse strains compared to the corresponding sham (Table 1). The C57BL/6J strain responded equally to both MMA-SS and S-Cr with approximately a 9% increase in BAL PMN. The C57BL/6J had ~2% increase in lymphocytes following the exposures, which was greater than both exposed A/J groups. In contrast, the A/J mice had a significantly greater response to MMA-SS (~40% PMN) but responded similarly to the C57BL/6J following S-Cr exposure (~11% PMN). Of note, although treatment effects were found for increased lung macrophage/monocytes in both strains following MMA-SS exposure, no strain differences were found for this inflammatory parameter (data not shown).

### BAL findings 7 days after exposure: GMA-MS and GMA-SS

By 7 days post-exposure, LDH remained significantly elevated in both mouse strains exposed to GMA-MS welding fume (Table 2). At this time point, the A/J mice had significantly greater LDH levels compared to the C57BL/6J strain. As with the earlier time point, no indication of epithelial damage occurred with this fume. The %PMN remained significantly elevated only in the A/J strain at 7 days (~12%). The GMA-SS fume caused a greater LDH and %PMN response in the A/J compared to the C57BL/6J. At 7 days, this fume was again more toxic to the A/J compared to the MS fume. No strain differences were found for increased lymphocytes or macrophage/monocytes, although a mild elevation versus the corresponding shams was found for the GMA-SS groups (macrophage data not shown).

**Table 2:** Bronchoalveolar lavage parameters 7 days post-exposure

Mouse Strain	*n*	Exposure	%Albumin^§^	%LDH^§^	%Lymphocytes^||^	%PMN^||^
A/J	7	GMA-MS	90 ± 3	156 ± 10*^‡^	0.64 ± 0.16 (0 ± 0)	12 ± 1.2*^‡ ^(0 ± 0)
C57BL/6J	7	GMA-MS	105 ± 3	132 ± 3*	0.54 ± 0.13 (0.14 ± 0.07)	0.72 ± 0.3 (0.05 ± 0.05)
						
A/J	6	GMA-SS	115 ± 6	216 ± 16* ^†‡^	0.86 ± 0.18* (0 ± 0)	20 ± 1.2*^†‡ ^(0 ± 0)
C57BL/6J	7	GMA-SS	113 ± 4	148 ± 8*	0.8 ± 0.17 * (0.14 ± 0.07)	1.3 ± 0.8 (0.05 ± 0.05)
						
A/J	7	MMA-SS	145 ± 11*	144 ± 17*	1.8 ± 0.5* (0.1 ± 0.06)	12 ± 1.0*^†‡ ^(0.1 ± 0.1)
C57BL/6J	7	MMA-SS	115 ± 8	130 ± 5*^†^	1.0 ± 0.36* (0 ± 0)	1.6 ± 0.1*^† ^(0.07 ± 0.07)
						
A/J	5	S-Cr	137 ± 12*	111 ± 13	2.2 ± 0.46* (0.1 ± 0.06)	5.6 ± 0.6*^‡ ^(0.1 ± 0.1)
C57BL/6J	7	S-Cr	124 ± 8*	111 ± 7	0.44 ± 0.23 (0 ± 0)	0.22 ± 0.2 (0.07 ± 0.07)

*Significantly different from corresponding sham; ^† ^Between exposed groups of the same mouse strain; ^‡^Between mouse strains of the same exposure group (p < 0.05)

^§^LDH and albumin data are represented as % corresponding sham. ^||^Percentage from a differential cell count of > 300 cells with the corresponding sham % in parentheses.

*Note*. Data are means ± SE. Statistical comparisons were MMA-SS and S-Cr or GMA-MS and GMA-SS. Abbreviations are GMA-MS-gas metal arc-mild steel, GMA-SS-gas metal arc stainless steel, or MMA-SS-manual metal arc stainless steel welding fume; S-Cr-soluble chromium VI; LDH-lactate dehydrogenase; PMN-polymorphonuclear leukocytes

### BAL findings 7 days after exposure: MMA-SS and S-Cr

No strain differences were observed for LDH or albumin at 7 days post-exposure to MMA-SS fume, although both parameters remained significantly elevated in the A/J, only LDH remained increased in the C57BL/6J (Table 2). Both strains in the S-Cr-exposed groups had increased albumin but no strain differences were noted. Significant strain differences were evident for %PMN influx. MMA-SS caused a more pronounced effect in the A/J strain with ~12% PMN versus ~6% in the S-Cr-exposed mice, while only a mild elevation was found for the exposed C57BL/6J groups. No strain differences were found for increased macrophage/monocytes, but an unremarkable increase in number was found in the MMA-SS groups (data not shown).

### BAL findings 28 days after exposure: GMA-MS and GMA-SS

By 28 days, LDH and albumin had returned to baseline in all groups exposed to GMA-MS (data not shown). The only strain difference noted was the PMN response which remained at ~10% in the A/J mice versus ~1% in the C57BL/6J. The A/J mice continued to have significant lung cell death, lymphocyte and PMN influx associated with GMA-SS exposure, and these responses were ~1.2–2 fold greater in comparison to the GMA-MS fume. The A/J strain exhibited a 9 fold greater %PMN response compared to the C57BL/6J following exposure to GMA-SS.

### BAL findings 28 days after exposure: MMA-SS and S-Cr

At 28 days post-exposure, LDH and albumin were not significantly elevated in the MMA-SS or S-Cr exposed groups of either strain (data not shown). Only the MMA-SS-exposed A/J mice had significant PMN in the BAL, approximately 4.4%.

Of note, for all groups, no strain differences were found for any BAL parameter in the sham mice at any time point.

### BAL inflammatory cytokine and lung gene expression analysis

The first fraction BAL supernatant was used to determine the protein levels of interferon-γ (IFN-γ), interleukin-6 (IL-6), interleukin-10 (IL-10), interleukin-12p70 (IL-12p70), monocyte chemoattractant protein-1 (MCP-1), and tumor necrosis factor-α (TNF-α) at 2, 7, and 28 days post-exposure to MMA-SS fume or S-Cr. Protein levels of IL-10 and IL-12p70 were unaltered by either exposure (data not shown).

**Figure 1:**
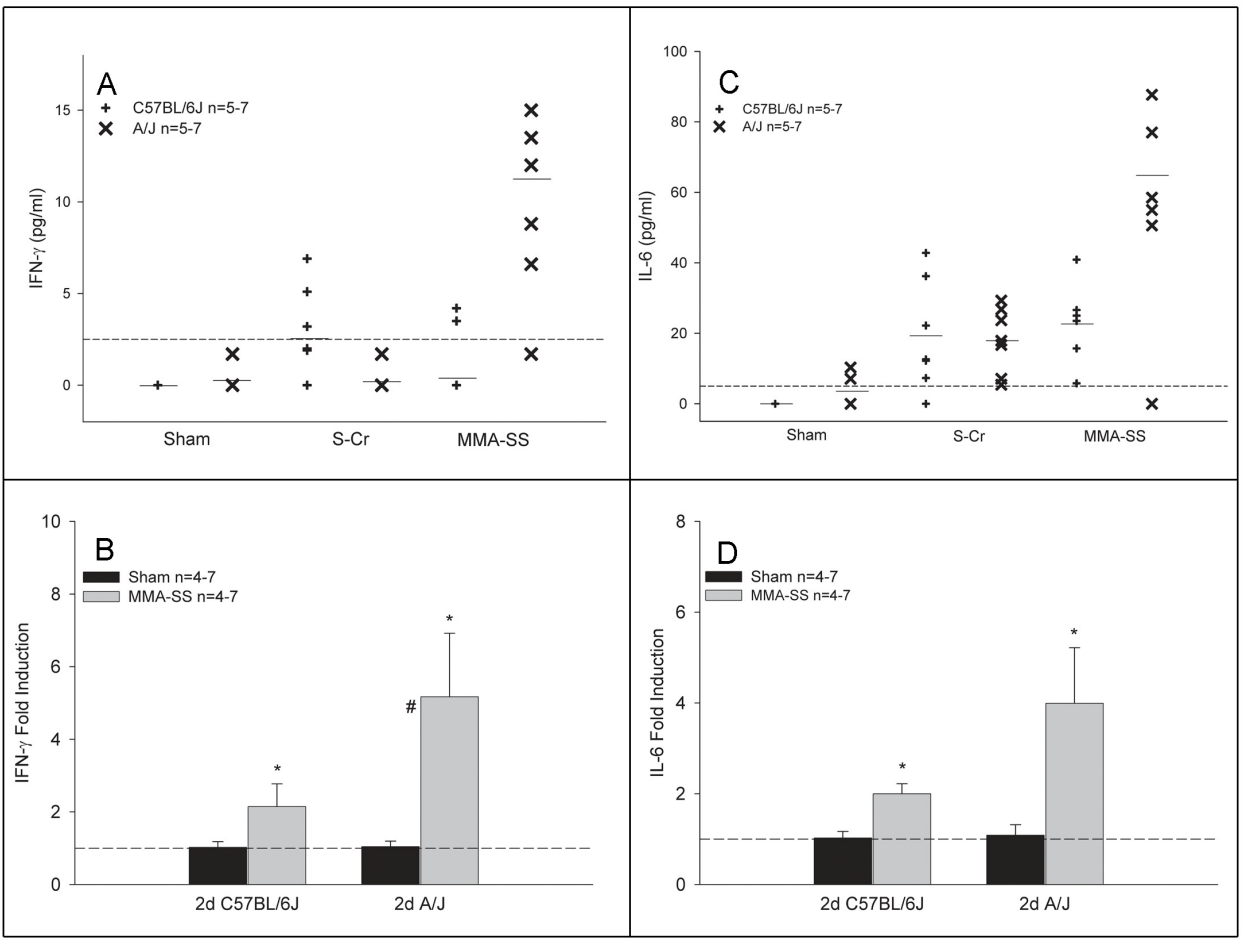
**Effect of S-Cr or MMA-SS welding fume on lavage protein levels and whole lung gene expression of IFN-γ (A&B) and IL-6 (C&D) at 2 days post-exposure in A/J and C57BL/6J mice.** The dotted line represents the assay sensitivity for each protein and mean lines (–) are shown for each group. Gene expression data are presented as fold change from sham controls (dotted line). Values are mean ± SE (n = 4–7 per group). *-Significantly different from corresponding sham. #-Significantly different between strains of the same exposure group *Note: Portions of this figure have been previously published *[42].

IFN-γ protein was increased following MMA-SS exposure in the A/J but not the C57BL/6J mice at 2 days (Figure 1A). By 7 days post-exposure, these levels remained moderately elevated (data not shown). No IFN-γ protein was detected in the C57BL/6J strain for either exposure at this time point. Whole lung gene expression confirmed these findings in the MMA-SS-exposed A/J mice. A mild increase in gene expression was found for the MMA-SS-exposed C57BL/6J mice but this was significantly less than levels found in the exposed A/J strain (Figure 1B). IL-6 protein was increased following S-Cr and MMA-SS exposure at 2 days in both strains (Figure 1C). The C57BL/6J responded equally to both exposures while the mean response to the MMA-SS was greater in the A/J mice. Gene expression confirmed these data as the A/J showed approximately a two fold greater increase in IL-6 mRNA compared to the C57BL/6J (Figure 1D). By 7 days, gene expression had returned to baseline and protein levels declined with a slight elevation remaining in the MMA-SS-exposed A/J strain (data not shown). By 28 days, protein levels were undetectable in all groups for both IFN-γ and IL-6 (data not shown). No differences were found between the two strains for gene expression levels in the shams at either time point.

**Figure 2:**
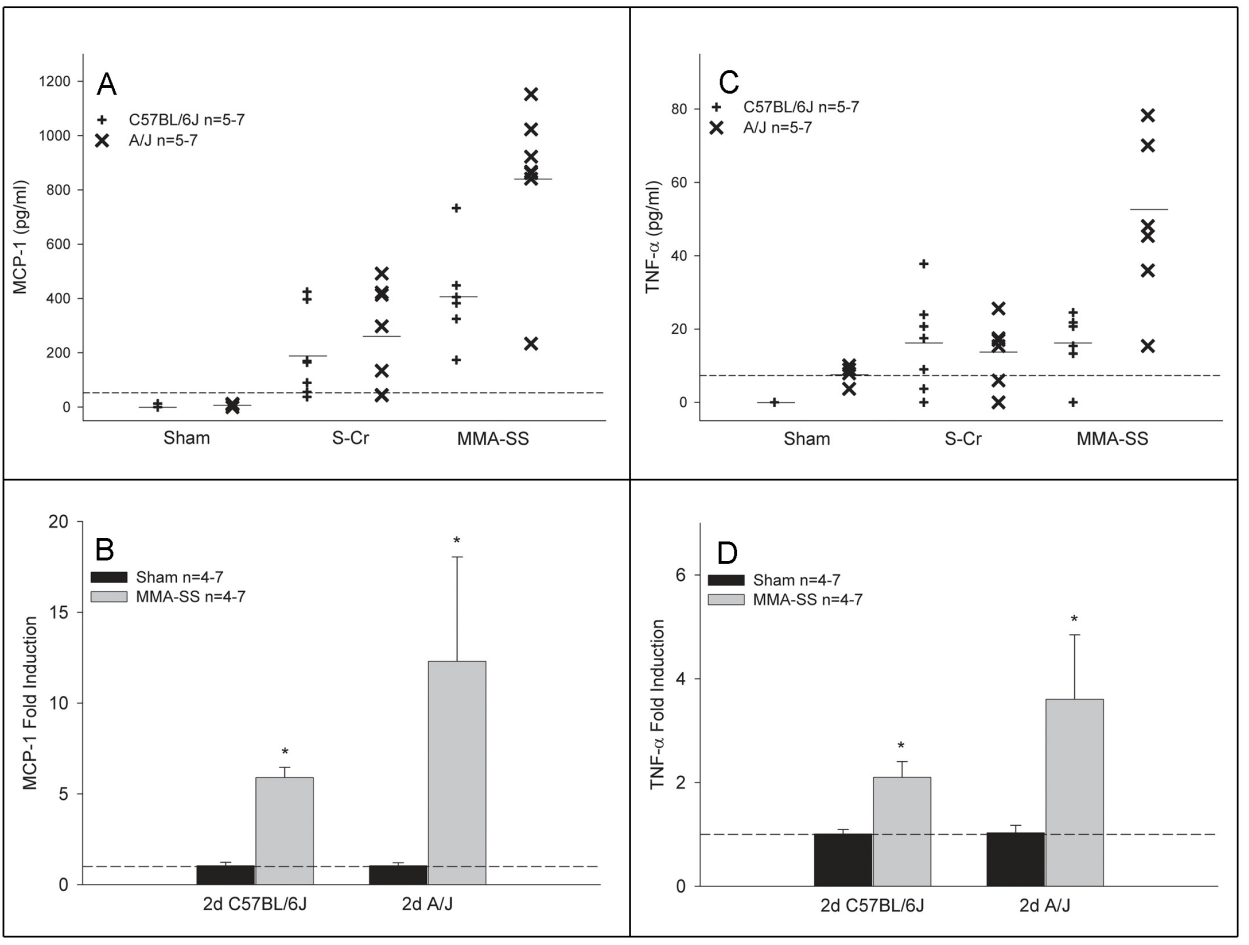
**Effect of S-Cr or MMA-SS welding fume on lavage protein levels and whole lung gene expression of MCP-1 (A&B) and TNF-α (C&D) at 2 days post-exposure in A/J and C57BL/6J mice.** The dotted line represents the assay sensitivity for each protein and mean lines (–) are shown for each group. Gene expression data are presented as fold change from sham controls (dotted line). Values are mean ± SE (n = 4–7 per group). *-Significantly different from corresponding sham. *Note: Portions of this figure have been previously published *[42].

A similar trend was found for protein and gene expression levels of MCP-1 and TNF-α (Figure 2). The A/J mice consistently had a greater response to MMA-SS compared to S-Cr and the C57BL/6J strain at 2 days post-exposure (Figure 2A &2C). Gene expression for MCP-1 and TNF-α revealed at least a two fold induction difference between the strains (Figure 2B &2D). By 7 days, gene expression returned to baseline while protein levels decreased but remained mildly elevated in the exposed A/J mice (data not shown). By 28 days, protein levels were undetectable in all groups for MCP-1 and TNF-α (data not shown). In addition, no differences were found between the two strains for gene expression levels in the saline shams at either time point.

**Figure 3:**
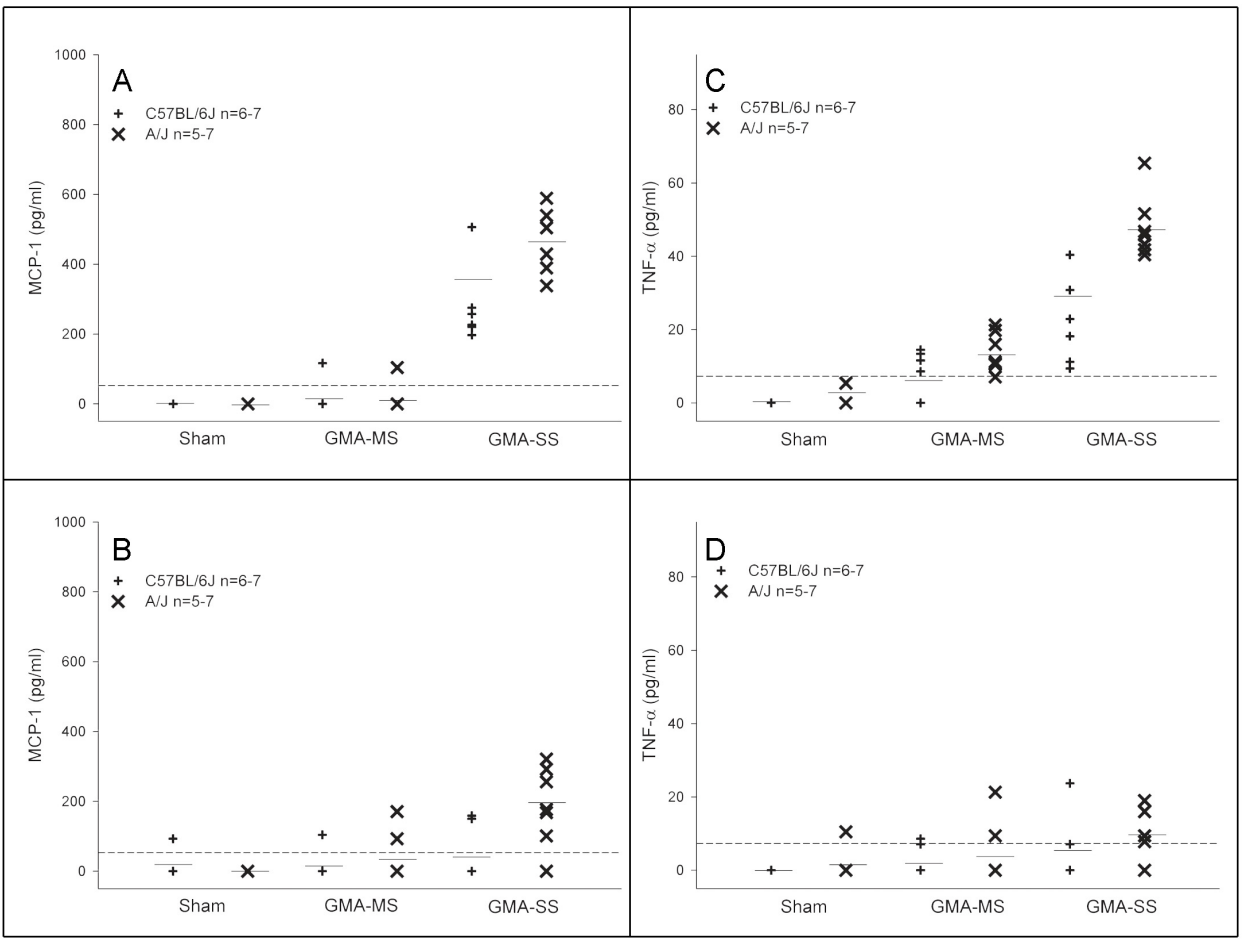
**Effect of GMA welding fumes on lavage protein levels of MCP-1 (A&B) and TNF-α (C&D) at 2 and 28 days post-exposure in A/J and C57BL/6J mice.** The dotted line represents the assay sensitivity for each inflammatory protein and mean lines (–) are shown for each group (n = 5–7).

The first fraction BAL supernatant was used to determine the protein levels of IFN-γ, IL-6, IL-10, IL-12p70, MCP-1, and TNF-α at 2, 7, and 28 days post-exposure to GMA welding fumes. Data are shown only for MCP-1 and TNF-α at the 2 and 28 day time points (Figure 3A–D); the IFN-γ, IL-6, and 7 day data are described below. No trends were found in either strain for IL-10 and IL-12p70 protein (data not shown).

IFN-γ protein was increased following GMA-SS fume exposure (9.0 ± 2.4 pg/ml) only in the A/J at 2 days post-exposure but was undetectable by day 7. IL-6 protein was greater in the GMA-SS-exposed A/J compared to the C57BL/6J at 2 days (23.3 ± 1.8 and 15.5 ± 5.3 pg/ml, respectively) and levels remained elevated at 7 days in the A/J (12.5 ± 2.6 pg/ml). By 28 days, IL-6 levels were undetectable in the A/J. GMA-MS exposure did not increase IFN-γ or IL-6 in either strain at any time point post-exposure (data not shown).

MCP-1 protein levels were increased following GMA-SS exposure at 2, 7, and 28 days in the A/J strain while the C57BL/6J only had elevated levels at day 2 (Figure 3A &3B). GMA-MS fume did not affect BAL levels of MCP-1 at any time point in either strain. TNF-α levels in the GMA-SS-exposed A/J were elevated at 2 (Figure 3C) and 7 days post-exposure and remained mildly elevated at 28 days (Figure 3D). The C57BL/6J had lower TNF-α levels at 2 and 7 days post-exposure and no detectable levels by 28 days. At 2 (Figure 3D) and 7 days, GMA-MS fume did increase BAL TNF-α levels in the A/J strain, although levels were lower in comparison to GMA-SS.

### Lung oxidative stress

No increased gene expression was found for prostaglandin-endoperoxide synthase 2 (COX-2), nitric oxide synthase 2 (iNOS or NOS2), or glutathione-*S*-transferase Pi (GST-Pi) at the 2 day time point for either mouse strain exposed to MMA-SS fume (data not shown). S-Cr samples were not analyzed because no strain differences were found by other parameters in this study. At 28 days, GMA or MMA-SS fumes did not cause increased expression of GST-Pi or COX-2 genes in the A/J (data not shown). Gene expression in the C57BL/6J was not analyzed at 28 days as the lung response of this strain had resolved by this time point (data not shown).

### Effects on body weight and survival 48 and 78 weeks after welding fume exposure

Fluctuations in body weight (± 1–2 g) occurred in all groups, but no effects on final body weight were observed due to welding fume exposure (data not shown). At the 48 and 78 week sacrifices, respectively, mice had gained an average (± SE) of 7.3 ± 0.63 g and 7.6 ± 0.20 g over the course of the study.

Survival was not different between any welding fume-exposed group and shams at either time point. At 48 weeks post-exposure, survival was > 91% for all groups. At 78 weeks, survival was 80% for the sham, GMA-MS and MMA-SS groups, and 73% for the GMA-SS group.

### Urethane positive control findings

Urethane exposed A/J mice had an average lung tumor multiplicity of 17.2 ± 0.97 (tumors/lung, n = 24) and 100% (24/24) incidence at 48 weeks. The survival rate was 96% at 48 weeks post-exposure. Due to the observed carcinogenic potency of urethane the 78 week group was sacrificed at 54 weeks. Survival had decreased to 85% by this time point post-exposure. Tumor multiplicity and incidence was 15.41 ± 1.64 (tumors/lung, n = 17) and 94% (16/17 mice), respectively.

### Gross tumor findings in A/J mice 48 and 78 weeks after welding fume exposure

At 48 weeks post-exposure, lung tumor multiplicity and incidence was greatest in the GMA-SS-exposed group (0.45 ± 0.14 and 40%, respectively) (Table 3). However, at this time point, statistical significance was not achieved. Shams had a 33% tumor incidence and a 0.38 ± 0.13 multiplicity upon gross exam. Increases in lung tumor incidence or multiplicity following exposure to GMA-MS or MMA-SS welding fumes were unremarkable. Average tumor size was ≤ 2 mm for all groups at 48 weeks.

**Table 3:** Gross lung tumor findings for A/J mice 48 and 78 weeks post-exposure

Exposure	Lung Tumor Multiplicity*		Lung Tumor Incidence^†^	
	48 wk	78 wk	48 wk	78 wk
Sham	0.38 ± 0.13 (21)	1.00 ± 0.35 (19)	33% (7/21)	53% (10/19)
GMA-MS Gas metal arc-mild steel	0.42 ± 0.14 (24)	1.00 ± 0.22 (20)	33% (8/24)	65% (13/20)
GMA-SS Gas metal arc-stainless steel	0.45 ± 0.14 (20)	1.75 ± 0.32 (16)	40% (8/20)	81% (13/16)
MMA-SS Manual metal arc-stainless steel	0.25 ± 0.11 (24)	1.55 ± 0.34 (20)	21% (5/24)	80% (16/20)

*Average number of tumors per lung (± SE) and includes mice with no tumors. Parentheses indicate total animal number. †Percentage of tumor-bearing mice out of the total. Parentheses indicate tumor-bearing/total animal number.

At 78 weeks, exposure to both SS welding fumes increased tumor multiplicity and incidence compared to sham and the GMA-MS fume, but this increase did not reach statistical significance (Table 3). However, gross tumor incidence between the GMA-SS and sham groups was 81% and 53%, respectively, and approached significance (p = 0.057). The tumor multiplicity in the GMA-SS was greater in comparison to all groups and was 1.75 ± 0.32 versus 1.00 ± 0.35 in the sham. No significant differences in tumor multiplicity or incidence were found for the MMA-SS or GMA-MS-exposed groups versus sham control. Average tumor size varied between 2.4–3.5 mm at 78 weeks, and was not different among exposure groups.

### Lung histopathological findings in A/J mice 48 and 78 weeks after welding fume exposure

**Figure 4:**
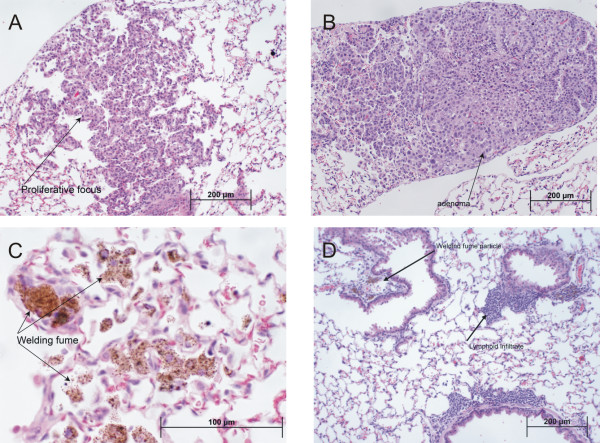
**Photomicrographs of lung tissue from welding fume-exposed A/J mice.** Preneoplastic lesions (panel A) and adenomas (panel B) were the most common lung lesions observed in all exposed and sham groups. These representative low magnification photomicrographs were captured at 48 weeks post-exposure to GMA-SS fume. Panel C is a high magnification photomicrograph showing the presence of GMA-SS fume (brown-black granular pigmented areas) at 48 weeks post-exposure. An increased persistence in the lung was observed for GMA-SS fume compared to the GMA-MS or MMA-SS fume. At 78 weeks post-exposure, the presence of GMA-SS fume is still observed in the lung and an increased lymphohistiocytic infiltrate was associated with this fume (panel D).

In addition to the gross tumor evaluation at necropsy, histopathological analysis was done on separately embedded lung lobes from all mice in the study to evaluate morphological changes (Table 4). At 48 weeks post-exposure, all lung lesions observed were either adenomas or preneoplastic epithelial proliferations (Figure 4A &4B). The GMA-SS group had the greatest increase in preneoplasia/tumor multiplicity and incidence (0.75 ± 0.15 and 65%). This was significantly different compared to the GMA-MS group (33%), but not sham (50%). Significance for incidence between the MMA-SS versus GMA-SS groups was borderline (p = 0.06). A significant increase in welding fume-containing macrophages was found in all exposed groups. GMA-SS welding fume was consistently found, in moderate amounts (3.03 ± 0.13), in all five lung lobes of all 20 mice evaluated whereas MMA-SS and GMA-MS fumes were variable and minimal in the lung, 0.67 ± 0.13 and 0.34 ± 0.10, respectively (Figure 4C). The presence of perivascular/peribronchial lymphoid infiltrates in the lung was not a significant finding at 48 weeks post-exposure to welding fume.

**Table 4:** Lung histopathology for A/J mice 48 and 78 Weeks post-exposure

	Exposure	n	Lymphoid Infiltrates*	Welding Fume-Laden Cells	Lung Tumor Multiplicity^†^	Lung Tumor Incidence^‡^
48 wk	Sham	21	1.08 ± 0.16	0 ± 0	0.55 ± 0.14	50%
	GMA-MS	24	0.30 ± 0.10	0.34 ± 0.10^§^	0.38 ± 0.12	33%
	GMA-SS	20	1.23 ± 0.22	3.03 ± 0.13^§^	0.75 ± 0.15	65%**
	MMA-SS	24	0.90 ± 0.16	0.67 ± 0.13^§^	0.38 ± 0.12	33%
						
78 wk	Sham	19	1.53 ± 0.29	0 ± 0	1.47 ± 0.33	68%
	GMA-MS	20	0.78 ± 0.24	0.15 ± 0.07^§^	1.40 ± 0.32	75%
	GMA-SS	16	2.53 ± 0.36^§^	1.12 ± 0.17^§^	1.94 ± 0.38	88%
	MMA-SS	20	1.70 ± 0.27	0.55 ± 0.19^§^	1.85 ± 0.46	75%

*Perivascular/peribronchial associated lymphocytes, macrophages, and plasma cells.

^†^Average number of tumors/preneoplastic lesions per lung, includes mice with no lesions.

^‡^Percentage of tumor/preneoplasia-bearing mice out of the total. ^§^Significantly increased compared to sham (p < 0.05). ^||^Significantly increased compared to GMA-MS and MMA-SS welding fumes (p < 0.05). **Significantly increased versus GMA-MS (p ≤ 0.05).

*Note*: Abbreviations are GMA-MS-gas metal arc-mild steel, GMA-SS-gas metal arc stainless steel, or MMA-SS-manual metal arc stainless steel welding fume. Data are mean ± SE.

By 78 weeks, tumor/preneoplastic lesions were observed in lungs of all groups and primarily were preneoplastic epithelial proliferations, adenomas, and adenomas arising within proliferations (Table 4). Carcinomas arising in an adenoma, carcinomas, and microcarcinomas were also observed but were less common. Again, multiplicity and incidence was greatest in the GMA-SS group, 1.94 ± 0.38 and 88%, respectively. This was not statistically different compared to multiplicity and incidence in the shams, 1.47 ± 0.33 and 68%, respectively. There was no association noted between the presence of any welding fume and preneoplastic or neoplastic lesions in the lung at 78 weeks after exposure.

The GMA-SS group also exhibited a significant increase in perivascular/peribronchial associated lymphoid infiltrates – composed of lymphocytes, macrophages, and plasma cells – at 78 weeks post-exposure (Figure 4D). These lung infiltrates, however, were not necessarily associated with the presence of GMA-SS fume particles in the lung. GMA-SS welding fume was present in most lung lobes of all the mice in that group and in twice the amount, as shown by the increased presence of welding fume-laden cells, compared to the other fumes. The GMA-MS and MMA-SS fumes were rarely observed in the lung at 78 weeks post-exposure.

## Discussion and conclusion

This study compared the lung response in lung tumor susceptible (A/J) and resistant (C57BL/6J) mice exposed to GMA-MS, GMA-SS, MMA-SS, and S-Cr and found notable strain-dependent differences with regards to the degree and resolution of the inflammatory response. We also found supportive, but not conclusive, evidence for a possible tumorigenic effect of GMA-SS welding fume in the lung tumor susceptible A/J mouse. The results of this study, however, did not suggest a tumorigenic effect of MMA-SS or GMA-MS welding fumes. To our knowledge, this is the first *in vivo *animal study to provide preliminary evidence for a possible association between welding fume exposure and lung tumorigenesis.

The A/J mouse model was chosen based on its similarities with human lung adenocarcinoma development-specifically, the anatomy, histogenesis, and molecular features [12]. For example, the pulmonary adenoma susceptibility (*Pas1*) locus, a major mouse lung tumor susceptibility locus first mapped in A/J mice, appears to have a human counterpart which also conveys susceptibility to the development of human lung adenocarcinoma [14,15]. These data suggest that findings in the welding fume-exposed A/J mouse lung tumor model may have direct relevance to humans. In addition, although welding fume-induced lung toxicity has been investigated using other rodent models, the direct comparison of a lung tumor susceptible versus resistant mouse strain will help to elucidate a relationship between lung inflammation and possible welding fume-induced tumorigenesis.

Stainless steel electrodes used during MMA or GMA welding generate fumes containing carcinogenic Cr and Ni. A major difference between the MMA- and GMA-generated fumes is the solubility and, therefore, possibly the bioavailability of the metals contained in the fumes. Indeed, regardless of fume generation, stainless steel fumes reportedly are toxic and mutagenic to mammalian cells [16,17], cause DNA strand breaks *in vitro *and increased apoptosis *in vivo *[18], and induce lung cell hyperplasia and atypia in mice [19]. Furthermore, previous data showed the soluble fraction of MMA-SS, which is abundant in Cr, generated free radicals and caused significant lung macrophage toxicity [9,20]. Therefore, S-Cr was used to investigate the acute lung toxicity of this metal, which has been implicated as the main reactive component of MMA-SS fume [10].

In the C57BL6/J strain, S-Cr appeared to be the main pneumotoxic component as the markers of lung injury and inflammation were not markedly different when compared to exposure to MMA-SS fume at all time points post-exposure. Conversely, the A/J exhibited a greater lung response to MMA-SS, which suggests other fume components such as the insoluble Ni, Mn, and Fe, in addition to S-Cr, exert toxic lung effects. Because chromium, in its hexavalent form, rapidly penetrates lung cells and is detoxified as it travels through the body [21,22], it is possible that an earlier time post-exposure may have revealed a response similar to the C57BL/6J in the A/J strain. In rats, previous time course data showed lung cytotoxicity and damage caused by intratracheal exposure to the insoluble or soluble fraction, or the total MMA-SS fume were similar at 3 hours and 1 day, but by 3 days the total fume caused additive effects in the lung compared to the separate fractions [20]. This divergent response in the rat appears to resemble that of the A/J, but not the C57BL/6J, in this study. Taken together, these data suggest that interactions between the fume fractions are an important feature of MMA-SS lung toxicity, at least in Sprague-Dawley rats and A/J mice.

Repeated exposure to the insoluble GMA-MS fume revealed a strain-dependent neutrophil influx (A/J > C57BL/6J) that persisted through 28 days. It appears the insoluble components in welding fume – predominantly Fe and Mn – may impact recruitment of lung PMN in A/J mice, a phenomenon also observed in rats [20]. GMA-MS exposure also caused slightly greater cytotoxicity in the A/J, apparent only at 7 days, but no lung damage in either strain. In agreement, intratracheal instillation of MS welding fume in rats, at a single bolus dose of 8 mg/kg, was associated with the least lung toxicity when compared to GMA-SS and MMA-SS fumes [20]. The magnitude of the lung response to GMA-SS fume in the A/J was, overall, also greater compared to the C57BL/6J strain. In contrast to the GMA-MS fume, significant lung cell death and epithelial damage were observed in both strains, but the lung effects of the GMA-SS fume were greater, and the recovery period longer, in the A/J strain. This suggests the GMA-SS fume may cause a more chronic lung response likely related to its increased persistence compared to the GMA-MS [23] and the possible sensitivity of the A/J strain to the insoluble Cr and Ni metal components of this fume.

For further confirmation of the BAL findings, key cytokines, reportedly involved in welding fume-induced lung inflammation, were evaluated [20]. The cytokine response profiles, both gene expression and protein measurements, mirrored the BAL profiles and revealed that the lung response to aspirated MMA-SS and S-Cr involved the pro-inflammatory cytokines IL-6, MCP-1, and TNF-α in both strains. Similarly, IL-6, MCP-1, and TNF-α were common between the strains after GMA-SS fume exposure and the response profiles also reflected that of the BAL. Again, GMA-SS exposure appeared to cause a prolonged effect in the A/J, evidenced by the persistent MCP-1 levels, which may indicate an ongoing macrophage migration to the lung. GMA-MS fume selectively increased lung TNF-α levels in the A/J mice only, further suggesting that, in comparison to the other welding fumes, it is associated with less lung activation. Of interest was the differential induction of IFN-γ, a potent macrophage-activating T helper 1 (Th1) cytokine. This cytokine was solely induced by the SS fumes and only in the A/J strain. No measurable IFN-γ protein was detected in the MMA-SS-exposed C57BL/6J but a modest increase in gene expression was detected. IFN-γ is produced exclusively by Th1 CD4 and CD8 cytotoxic T lymphocyte (CTL) effector cells and natural killer (NK) cells. Its antiproliferative, antitumor, and immunomodulatory properties have been extensively reviewed [24]. The relevance of our finding that IFN-γ is selectively increased following SS welding fume exposure – primarily in the A/J – is unclear. However, it may suggest a role for cytotoxic T cells and NK cells in the immune response to SS welding fume in the A/J.

The recovery of the A/J lung response to welding fume, assessed via BAL, was attenuated when compared to the C57BL/6J strain. In fact, at 78 weeks after exposure, histopathology of the A/J lung showed that GMA-SS welding fume continued to exert significant, but mild, inflammatory lung effects, i.e. lung lymphoid infiltrates, which may be correlated with its increased biopersistence compared to the other fumes. In regards to tumorigenic effects, the insoluble Cr and Ni components of this fume may be of even greater importance given that the insoluble, iron-abundant, GMA-MS and soluble chromium-containing MMA-SS fume showed no consistent trends for incidence or multiplicity in this mouse model. In fact, negative or weak effects of soluble chromium on *in vivo *tumorigenicity has been reported which seems to be consistent with our preliminary results [21,25]. Further study is necessary to completely understand the importance of our current observations, but support exists for a relationship between genetic susceptibility to lung tumorigenesis and inflammation. To date, the mechanism(s) remain unknown by which *Pas1 *confers lung tumor susceptibility. Anti-inflammatory drugs have been shown to inhibit tumorigenesis in A/J mice, which suggests inflammation and tumorigenesis may be linked [26]. Our long term preliminary findings which showed gross tumor incidence, but not multiplicity, to be nearly significant (p = 0.057), when considered in conjunction with the BAL results and histopathology, suggest that a chronic lung response to GMA-SS welding fume may enhance tumorigenesis in the A/J model. Certainly, more animal studies are needed to clearly define if welding fume causes lung tumorigenesis and which metal constituents, or combination thereof, play a greater role.

It is reported that A/J mice begin to develop spontaneous pulmonary tumors at 12–16 weeks of age [27]. Grossly observed background tumor frequency, as reported in the literature, can range from 31–40% between 43–53 weeks of age and increase to 65% by approximately 66 weeks [28-30]. Microscopically, adenomas and proliferations are the most commonly observed pathologies of both spontaneous and chemically-induced lung lesions in the A/J mouse [31]. Our preliminary results regarding tumor frequency and pathology are largely in agreement with those stated in the literature. We observed that the majority of the lung lesions in the sham and exposed groups were preneoplastic epithelial proliferations, adenomas, and/or adenomas arising within proliferations. In humans, lung cancers are more histologically diverse compared to the mouse, and adenocarcinomas are the more common diagnosis [32]. In A/J mice, the production of lung adenomas in is in fact relevant to the production of lung adenocarcinomas in humans as it appears that lung adenomas are the direct precursor to lung adenocarcinomas. Furthermore, in most cases, human and A/J mouse lung tumors both originate from atypical hyperplastic foci in the lung periphery [33-36]. Our observed background tumor frequency was 33% at 55 weeks of age and 50% at 85 weeks upon gross exam. Although this model is useful in many respects, the spontaneous tumor rate in the A/J model is an obvious limitation and, when combined with the observed mortality in this study, reduced our statistical power for detecting differences to only 50%. However, the tendency for the GMA-SS group to consistently have the greatest lung tumor incidence and multiplicity – both histopathologically and grossly observed – is difficult to ignore. Although this fume does not appear to be an exceedingly potent carcinogen at our cumulative exposure dose of 340 μg, which is representative of an approximately 196 day exposure in a 75 kg human, further investigation is indeed warranted. Currently, inhalation studies are ongoing using our recently developed automated robotic welder [37] with significantly larger group sizes to better control for the background incidence and to confirm our preliminary results with the GMA-SS fume.

Welding generates a complex mixture of particles and gaseous by-products; thus, it is not remarkable that notable differences are observed between the lung response following instillation of the fume and inhalation [38]. In fact, it is shown that freshly generated SS welding fumes are more biologically reactive compared to "aged" fumes, such as those used in this study [39]. This decreased reactivity may explain why there was no increased gene expression for the selected markers of oxidative stress in this study. Inhalation also more adequately mimics an occupational exposure, and daily exposures in the automated welder will allow for a greater and more gradual accumulation of fume in the mouse lung. Such an exposure is difficult to achieve with pharyngeal aspiration as repeated exposures are labor intensive in a study with such large group sizes. Therefore, it is apparent that the ongoing inhalation studies may offer more insight into the toxicity and tumorigenicity of welding fumes.

## Methods

### Animals

Male C57BL/6J and A/J mice, 4 weeks of age were purchased from Jackson Laboratories (Bar Harbor, ME) and housed in an AAALAC-accredited, specific pathogen-free, environmentally controlled facility. All mice were free of endogenous viral pathogens, parasites, mycoplasmas, Helicobacter, and CAR Bacillus. Mice were individually housed in ventilated cages and provided HEPA-filtered air under a controlled light cycle (12 hour light/12 hour dark) at a standard temperature (22–24°C) and 30–70% relative humidity. Animals were acclimated to the animal facility for a minimum of one week and allowed access to a conventional diet (6% Irradiated NIH-31 Diet, Harlan Teklad, Madison, WI) and tap water *ad libitum*. All procedures were performed using protocols approved by the National Institute for Occupational Safety and Health Institutional Animal Care and Use Committee.

### Welding fume collection and characterization

The welding fumes used in this study were provided by Lincoln Electric Co., (Cleveland, OH). The collection and characterization of these fumes were previously described [9]. Briefly, the fumes were generated in a cubical open-front fume chamber (volume = 1 m^3^) by a skilled welder, using a manual or automatic technique appropriate for the electrode, and then collected on a sterile 0.2 μm filter. The samples were generated by three welding processes: gas metal arc welding (with argon and CO_2 _shielding gases) using a mild steel electrode; gas metal arc welding using a stainless steel electrode; and manual metal arc welding using a flux-cored stainless steel electrode. Reported in Table 5 are the metal constituents, solubility/insolubility ratio, and pH of each welding fume sample [9]. Seven different metals (Cr, Cu, Fe, Mn, Ni, Ti, and V) commonly found in welding fumes were measured using inductively coupled argon plasma atomic emission spectroscopy. Count mean diameters were 1.22, 1.38, and 0.92 μm for the GMA-MS, GMA-SS, and MMA-SS samples, respectively, as determined by electron microscopy [9].

**Table 5:** Welding fume characterization by ICP-AES*

Welding Fume Sample	Metal (weight %)^†^	Soluble/insoluble Ratio	pH
GMA-MS Gas metal arc-mild steel	Fe 85 Mn 14	0.020	Total 7.02 Soluble 7.44 Insoluble 7.03
			
GMA-SS Gas metal arc-stainless steel	Fe 53 Mn 23 Cr 19 Ni 5	0.006	Total 6.94 Soluble 6.97 Insoluble 7.01
			
MMA-SS Manual metal arc-stainless steel	Fe 41 Cr 29 Mn 17 Ni 3	0.345 Soluble metals: Cr 87% Mn 11%	Total 6.92 Soluble 7.05 Insoluble 7.09

*Inductively Coupled Argon Plasma Atomic Emission Spectroscopy. † Relative to all metals analyzed. *Note*. Abbreviations are Cr-chromium; Fe-iron; Mn-Manganese; Ni-nickel. Data presented are referenced from Antonini et al., 1999.

### Welding fume and soluble chromium preparation

Each welding fume was weighed and suspended in sterile Ca^+2 ^and Mg^+2^-free phosphate buffered saline (PBS) in a 50 ml sterile conical tube. Following the initial preparation, the fume samples were vortexed then sonicated for 1 minute using a Sonifier 450 Cell Disruptor (Branson Ultrasonics, Danbury, CT). Prior to dosing, the samples were vortexed then sonicated for 15 seconds and vortexed immediately before each mouse exposure. For each experimental time point, fresh welding fume suspensions were made and the same preparation was used to expose both strains of mice.

Soluble chromium in the form of sodium dichromate dihydrate (Na_2_Cr_2_O_7_·2H_2_O) (Sigma-Aldrich, St. Louis, MO) was used to represent the hexavalent chromium species found in the MMA-SS fume as shown in previous studies [20,40]. For each experimental time point, fresh Na_2_Cr_2_O_7_·2H_2_O was weighed into a sterile 50 ml conical tube, suspended in sterile PBS then vortexed. The same preparation was used to expose both strains of mice.

### Mouse pharyngeal aspiration exposure

Age and weight-matched A/J and C57BL/6J mice were exposed to GMA-MS, GMA-SS, MMA-SS, S-Cr, or sterile Ca^+2 ^and Mg^+2^-free PBS (vehicle control) by pharyngeal aspiration as previously described [41]. Briefly, each mouse was placed in a glass jar with a gauze pad moistened with isoflurane (Abbott Laboratories, North Chicago, IL) until slowed breathing was observed. The mouse was then suspended, by its top incisors, on a slanted board in a supine position. The tongue was extended with forceps and the solution was placed by pipette at the back of the throat. The tongue was held extended until the solution was aspirated into the lung and the mouse resumed a regular breathing pattern. When performed properly, this technique allows minimal sample loss to the digestive tract. The mouse was then returned to its cage to recover, typically 10–15 seconds.

In this study, mice were exposed over a 10 day period to four bolus doses of test material in lieu of a single bolus dose. This regime achieved an accumulation of particles in the lung over time, which may be more representative of an occupational exposure. Mice were exposed four times (once every 3 days) to 85 μg (~5 mg/kg) of GMA-MS, GMA-SS, or MMA-SS welding fume, or 25.5 μg (~1.5 mg/kg) S-Cr. The cumulative fume lung burden was derived from our previous pharyngeal aspiration experiment in the A/J mouse and is equivalent to ~196 days of exposure in a 75 kg welder working an eight hour shift [19]. The dose of S-Cr was equal to the weight % of Cr (~30%) found in the MMA-SS total suspension (Table 5). A 25 μl aspiration volume was used and shams were administered an equal volume of PBS. Mice were sacrificed 2, 7 and 28 days post the fourth exposure. For the tumor study, A/J mice (n = 25/group) were exposed using the same protocol to GMA-MS, GMA-SS, or MMA-SS welding fume and sacrificed 48 and 78 weeks after the fourth exposure. S-Cr was not included as a group in the tumor study because our main objective was to first examine the effects of welding fumes, not their components.

### Urethane exposure

Urethane (Acros Organics N.V., Fair Lawn, NJ, CAS#51-79-6) served as a positive control for confirmation of A/J tumor susceptibility in this study. A single i.p. dose of urethane (0.75 g/kg) was administered to two independent A/J vendor lots. The positive control group was run in parallel with the control and welding fume-exposed groups for both the 48 week (n = 25) and 78 week (n = 20) time points.

### Body weight determination

For the comparative strain study, mice were weighed after the one week acclimation period, throughout the dosing, and again at the 2, 7, and 28 day sacrifices. The average body weight after the final exposure was approximately 17 ± 0.53 g for both mouse strains. All groups gained weight throughout the study and no treatment effects were observed.

A/J mice kept for 48 and 78 weeks post-exposure were weighed after the one week acclimation period, during dosing, and every 4 weeks until sacrifice. The average body weight (± SE) at the start of the study for the sham and welding fume groups was 18.9 ± 0.17 g and 18.4 ± 0.20 g for the 48 and 78 week groups, respectively.

### Bronchoalveolar lavage

BAL of the whole lung was used to assess lung inflammation and injury at 2, 7, and 28 days post-exposure to various welding fumes or S-Cr in A/J and C57BL/6J mice. Mice were deeply anesthetized with Sleepaway [26% sodium pentobarbital, 7.8% isopropyl alcohol and 20.7% propylene glycol] (Fort Dodge Animal Health, Fort Dodge, IA) then weighed. Once unresponsive, the abdomen was opened and the vena cava was exsanguinated. For BAL, the trachea was cannulated with a blunted 22 gauge needle and, while massaging the thorax, 0.6 ml of cold PBS was slowly instilled into the lung then withdrawn and placed into a 15 ml conical tube. This constituted the first fraction BAL fluid. Two subsequent lavages (1.0 ml/instillate) were collected into a separate tube which represented the second fraction. The BAL fluid was preserved on ice until four animals were sacrificed then the samples were centrifuged (500 × *g*, 10 min, 4°C).

Aliquots of the first fraction BAL supernatant were used to assess lung injury or frozen at -80°C for later analysis. The supernatant of the second fraction was discarded. The cell pellets from both fractions were combined and centrifuged (500 × g, 6 minutes, 4°C) and the supernatant discarded. The final cell pellet was suspended in a known volume of PBS and used for cell enumeration and differential staining.

Total cell numbers were determined using a Coulter Multisizer II and AccuComp software (Coulter Electronics, Hialeah, FL). For differential staining, BAL cells were plated onto glass slides using a Cytospin 3 centrifuge (Shandon Life Sciences International, Cheshire, England) set at 800 rpm for 5 minutes. Slides were stained using Leukostat stain (Fisher Scientific, Pittsburgh, PA) then coverslipped. A minimum of 300 cells/slide consisting of alveolar macrophages/monocytes, lymphocytes, or PMN were identified using light microscopy. Slides from shams consisted typically of > 99% alveolar macrophages.

### Bronchoalveolar lavage fluid cytokine analysis

Concentrations of cytokines from the first fraction BAL supernatant were measured using a mouse inflammation cytometric bead array kit (BD Biosciences, San Diego, CA) and analyzed on a FACSCalibur flow cytometer as previously described [42]. The following cytokines were measured: IL-6, IL-10, MCP-1, IFN-γ, TNF-α and IL-12p70. Standard curves with a range of 20–5,000 pg/ml were determined for each cytokine. The sensitivity of the assay for each protein ranged from 2.5–52.7 pg/ml. Because BAL protein levels for some analytes were at or below assay sensitivity, which limited statistical analysis, confirmation of selected BAL profiles by real-time reverse transcription polymerase chain reaction (RT-PCR) [see below] was done [42]. To appropriately represent the protein data, a scatter plot was chosen with the mean values indicated for each group.

### Biochemical measurements

Albumin, a measure of damage to the lung alveolar epithelial barrier, and LDH activity, indicative of lung cell death, were measured in the first fraction BAL fluid supernatant. The albumin concentration was determined colorimetrically at 628 nm based on albumin binding to bromcresol green, using an albumin BCG diagnostic kit (Sigma Chemical Co., St. Louis, MO). LDH activity was determined by measuring the oxidation of lactate to pyruvate coupled with the formation of NADH at 340 nm. Both measurements were performed with a COBAS MIRA Plus auto-analyzer (Roche Diagnostic Systems, Montclair, NJ).

### Real-Time RT-PCR

In separate identical experiments, whole lungs were removed from sham and welding fume-exposed mice then snap frozen in liquid nitrogen and stored at -80°C for RNA isolation. RNA was isolated from whole lung homogenates using the TRIzol (Invitrogen, Carlsbad, CA) method and then cleaned according to the manufacturer's instructions using a RNeasy Mini Kit (Qiagen, Valencia, CA) to remove possible DNA contamination. One μg of total RNA was reverse-transcribed using random hexamers (Applied Biosystems, Foster City, CA) and Superscript II (Invitrogen, Carlsbad, CA). Five μl of cDNA (in duplicates for each gene) was then used for gene expression determination using the Applied Biosystems 7900HT (Foster City, CA). The ribosomal subunit 18S was used as the housekeeping gene (Hs99999901_s1, Applied Biosystems). Relative gene expression was calculated using the comparative threshold method (2-ΔΔCt) [43].

The cytokine bead array findings from the MMA-SS 2 and 7 day post-exposure time points only were verified using the following Pre-designed Assays-on-Demand™ TaqMan^® ^probes and primers from Applied Biosystems: IFN-γ (Mm00801778_m1), IL-6 (Mm00446190_m1), MCP-1 (Mm00441242_m1), and TNF-α (Mm00443258_m1).

The following oxidative stress markers were also evaluated using RT-PCR: NOS2 or iNOS [Mm00440485_m1], COX-2 [Mm00440485_m1], and GST-Pi [Mm00839138_g1].

### Gross tumor counts and histopathology

A/J mice were euthanized by carbon dioxide asphyxiation, weighed, then the abdomen was opened and the vena cava exsanguinated. The whole lung was excised and gross tumor counts and measurements were recorded for each lung lobe. Apparent merged tumors, defined as a single tumor pattern in double-nodule form or an apparent collision of two different tumors, were counted as one because this was impossible to distinguish at necropsy. The lungs were inflated and fixed with 10% neutral buffered formalin for a minimum of 24 hours. Each lung lobe (apical, azygos, cardiac, diaphragmatic, left) was separately embedded in paraffin then a 5 μm standardized section was cut from each lung lobe. Slides were stained with hematoxylin and eosin and interpreted by a contracted board certified veterinary pathologist in a blinded fashion for morphological changes and proliferative/neoplastic lesions. If abnormal changes were found, severity was scored as follows: 1 = minimal, 2 = mild, 3 = moderate, 4 = marked, 5 = severe. The final severity score reflects the average of the right and left lung lobe scores. Proliferative/neoplastic changes were scored as P = preneoplastic epithelial proliferation, AP = adenoma arising within a proliferation, A = adenoma, CA = carcinoma arising within an adenoma, C = carcinoma, or MC = microcarcinoma according to Belinksy *et al*. [33]. Since examination of a single histological section per lung underestimates the total number of lesions per lung [44], the gross count at necropsy would be more representative of the response. However, for completeness, both microscopic and gross exam were statistically evaluated in this study. Histopathological interpretation was not done on lung sections from urethane-exposed mice because this was previously reported and was not our purpose [45,46].

### Statistical comparisons and analysis

For the 2, 7, and 28 day study, the mouse exposures were designed to account for the following statistical comparisons: 1) MMA-SS versus S-Cr and 2) GMA-MS versus GMA-SS between the two mouse strains within a single time point. Therefore, the exposures for each experimental time point (2, 7, or 28 days post-exposure) were run in parallel with both strains and shams were run with every exposure. Statistical comparisons were not made between different time points post-exposure or between the MMA-SS and GMA welding fumes.

All analyses were performed either using JMP version 5.0.1, or the SAS system for Windows version 9.1 (SAS Institute, Cary, NC). Factorial analysis of variance (ANOVA) was utilized on continuous variables to incorporate strain and treatment into each analysis. For some variables a log transformation was performed on the data to reduce heterogeneous variance and meet the assumptions of the ANOVA. All post-hoc comparisons were carried out using Fishers Least Significant Difference Test. For all analyses, the criterion of significance was p < 0.05. Gross tumor counts and histopathology counts from sections were analyzed similarly. Tumor incidence (presence or absence of tumors) was analyzed using a Chi-Square test in SAS 'Proc Freq', while tumor multiplicity (number of tumors/lung) was analyzed using Poisson regression in SAS 'Proc Genmod'. All analyses on tumor data utilized only those animals surviving the complete 48 and 78 week time points.

## Competing interests

The authors declare that they have no competing interests.

## Authors' contributions

PCZE performed the animal exposures, bronchoalveolar lavage, isolated RNA, and drafted the manuscript. MLK conducted the statistical analyses. LAB counted the mouse lung tumors and assisted with the lung preparation for histopathology. SHY performed the CBA assays. AE performed the RT-PCR experiments. JRR participated in the 48 and 78 week study sacrifices. SHR, JMA, and PCZE conceived and designed the study. All authors read and approved the final manuscript.

## Disclaimer

The findings and conclusions in this report are those of the authors and do not necessarily represent the views of the National Institute for Occupational Safety and Health.
